# Phenotyping of cancer-associated somatic mutations in the BCL2 transmembrane domain

**DOI:** 10.1038/s41389-024-00516-3

**Published:** 2024-04-26

**Authors:** Diego Leiva, Estefanía Lucendo, Alicia Belén García-Jareño, Mónica Sancho, Mar Orzáez

**Affiliations:** Targeted Therapies on Cancer and Inflammation Laboratory, Centro de Investigación Príncipe Felipe, Valencia, Spain

**Keywords:** Cancer genetics, Tumour heterogeneity

## Abstract

The BCL2 family of proteins controls cell death by modulating the permeabilization of the mitochondrial outer membrane through a fine-tuned equilibrium of interactions among anti- and pro-apoptotic members. The upregulation of anti-apoptotic BCL2 proteins represents an unfavorable prognostic factor in many tumor types due to their ability to shift the equilibrium toward cancer cell survival. Furthermore, cancer-associated somatic mutations in *BCL2* genes interfere with the protein interaction network, thereby promoting cell survival. A range of studies have documented how these mutations affect the interactions between the cytosolic domains of BCL2 and evaluate the impact on cell death; however, as the BCL2 transmembrane interaction network remains poorly understood, somatic mutations affecting transmembrane regions have been classified as pathogenic-based solely on prediction algorithms. We comprehensively investigated cancer-associated somatic mutations affecting the transmembrane domain of BCL2 proteins and elucidated their effect on membrane insertion, hetero-interactions with the pro-apoptotic protein BAX, and modulation of cell death in cancer cells. Our findings reveal how specific mutations disrupt switchable interactions, alter the modulation of apoptosis, and contribute to cancer cell survival. These results provide experimental evidence to distinguish BCL2 transmembrane driver mutations from passenger mutations and provide new insight regarding selecting precision anti-tumor treatments.

## Introduction

BCL2 family proteins modulate mitochondrial membrane permeabilization, which is considered the central point of control in the cell death pathway [1, 2]. The pro-apoptotic members of the BCL2 family (BAX, BAK, and BOK) interact with the anti-apoptotic members (e.g., MCL1, BCL2, BCLxL, and A1) in a complex network that determines cell death or survival [3, 4].

Both overexpression and mutations affecting the anti-apoptotic protein BCL2 provide cancer cells with a survival advantage [5, 6], leading the scientific and pharmaceutical communities to invest significant efforts into developing drugs specifically targeting this protein [7–9]. For example, the specific BCL2 inhibitor Venetoclax recently reached the clinic for the treatment of chronic lymphocytic leukemia [10, 11].

Somatic mutations affecting the cytosolic domains of BCL2 correlate with poor prognosis and a loss of sensitivity to anti-tumor treatments, which has prompted the development of alternative therapeutic strategies for affected cancer patients [5, 12, 13]. The Catalog of Somatic Mutations in Cancer (COSMIC) (http://cancer.sanger.ac.uk) database reveals that specific cancer mutations located in the transmembrane domain (TMD) of BCL2 [14, 15]; while said mutations suffer from low frequency, they have been classified as potentially tumorigenic. The evaluation of their pathogenicity relies on predictions of the functional, molecular, and phenotypic consequences of amino acid substitutions using Hidden Markov models [16]; however, we currently lack experimental studies evaluating these mutations regarding their ability to induce changes in the anti-apoptotic function of BCL2.

The TMD interaction network of the BCL2 protein family and its relevance in controlling cell death remains incompletely understood due to the complexity of working with membrane proteins. Our group recently described that TMD interactions between BCL2 and the pro-apoptotic protein BAX (together with cytosolic interactions) contribute to the control of cell death [17]. Here, we perform an exhaustive study of somatic mutations impacting the BCL2 TMD by investigating their insertion capacity, the ability to hetero-interact with BAX, and the functional consequences on cell death. Our results prompted us to refine the classification of mutations impacting the BCL2 TMD and elucidate those potentially providing an advantage for tumor progression or therapy resistance. In summary, understanding the impact of BCL2 TMD mutations at the molecular and functional level remains critical to unraveling the underlying mechanisms driving tumor behavior and developing precision treatments for affected patients.

## Material and methods

### BCL2 somatic mutants

**Fig. 1:**
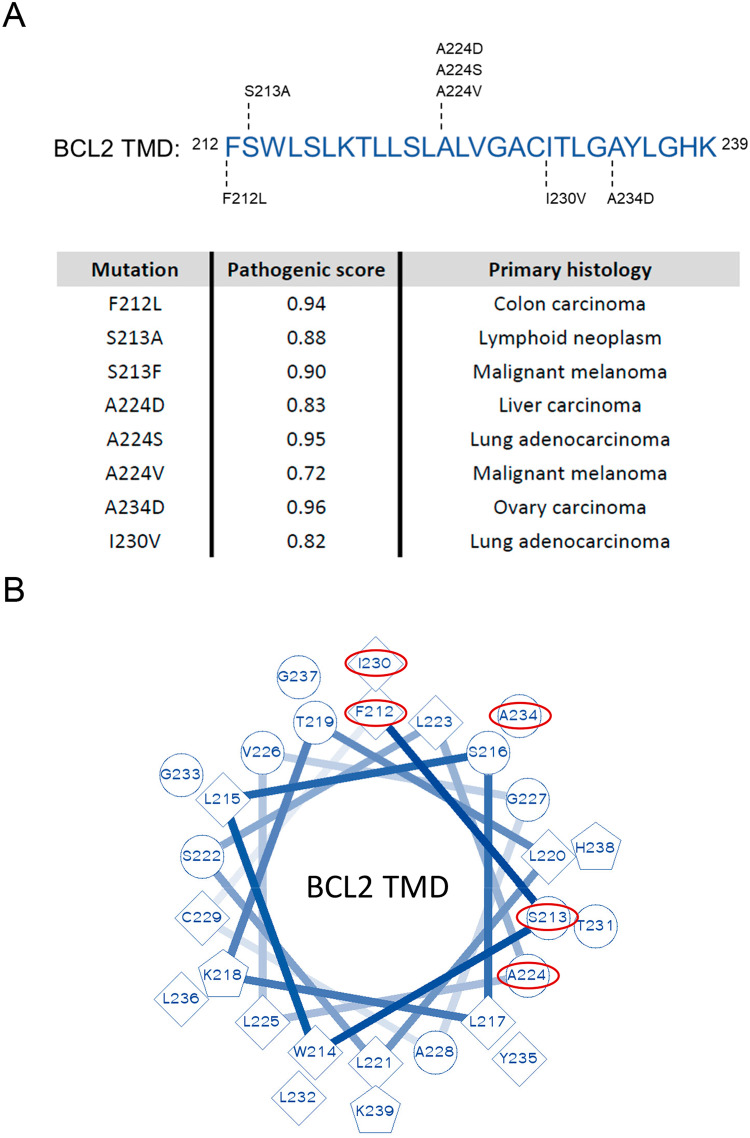
Mutations impacting the BCL2 transmembrane domain described in tumors. **A** Summary of mutations found in patients recorded in the COSMIC database, with tumor origin and pathogenic score provided. Scores ≤ 0.5 are considered neutral, and ≥0.7 are pathogenic. **B** Schematic representation of the impacted location of mutations (red circles) in a putative helical wheel conformation of the BCL2 TMD domain.

Somatic mutations that affect the BCL2 TMD were identified using the catalog of somatic mutations in cancer (COSMIC database) [18] (Fig. 1). BCL2 TMDs and BCL2 FL mutants were obtained from wild-type BiFC TMD constructs, and the pcDNA3.1/BCL2 vector using standard site-directed mutagenesis (Stratagene Quikchange II kit). Helical wheel projections were obtained with the web tool (https://pss.sjtu.edu.cn/cgi-bin/wheel.cgi?sequence).

### Cell culture

The HCT116 human colorectal carcinoma cell line was purchased from DSMZ (ACC581). Cells were grown in McCoy’s 5A medium supplemented with 10% fetal bovine serum. HeLa human ovarian cancer cells (CCL2; ATCC) were grown in DMEM supplemented with 10% fetal bovine serum. Cell lines were cultured in a humidified atmosphere of 5% CO_2_ and air at 37 °C.

### Bimolecular fluorescence complementation-TMD assays

BiFC analyses were performed as previously described [19]. Briefly, the BCL2 and BAX TMDs were cloned at the C-terminal end of the Venus VN-terminal (1-155, I152L) and VC-terminal (155-238, A206K). HCT116 cells were co-transfected with 0.5 μg of VN and VC DNA constructs using TurboFect (Thermo Scientific™). Fluorescence emission was measured after 16 h of transfection in phosphate-buffered saline (PBS) using 96-well black plates and a Wallac 1420 Workstation (*λ*_exc_ 510 nm and *λ*_em_ 535 nm). Transfection efficiency in HCT116 is controlled using the mCherry2-C1 plasmid (Addgene plasmid #54563). Flow cytometry studies showed that the percentage of cells incorporating both cherry control and BiFC plasmids corresponds to 60–70% of the population.

### Immunoblotting

Whole-cell extracts were subjected to sodium dodecyl-sulfate polyacrylamide gel electrophoresis (SDS–PAGE), transferred to nitrocellulose membranes, and blotted following standard procedures. Membranes were probed with the following primary antibodies: rabbit polyclonal anti-Tom20 (clone FL-145, no. 11415) from Santa Cruz; mouse monoclonal anti-c-myc (clone 9B11, no. 2276S) and rabbit monoclonal anti-HA (C29F4; no. 3724) from Cell Signaling and rat anti-tubulin YL1/2 (ab6160) from Abcam. Secondary antibodies specific for mouse, rabbit, or rat IgG conjugated with peroxidase for enhanced chemiluminescence (ECL) detection were purchased from Sigma-Aldrich. Immunoblot signals were detected using ECL detection reagents and acquired in an ECL™ Detection System (Roche).

### Subcellular fractionation and carbonate extraction

HCT116 cells transfected with BCL2 TMDs were harvested, washed in cold PBS, and resuspended in cold SEM buffer (10 mM HEPES and 250 mM sucrose, pH 7.2) supplemented with a complete protease inhibitor cocktail (Roche). Cells were mechanically lysed by passing them through a 23-gauge needle. Nuclei and cellular debris were removed by centrifugation at 500 × *g* for 5 min at 4 °C. The post-nuclear supernatant was centrifuged at 15,000 × *g* for 30 min at 4 °C, and the cytosol (C; supernatant) and heavy membranes (M; pellet) fractions were separated. The crude heavy membrane pellet was resuspended in 0.1 M cold sodium carbonate solution (pH 11.5), incubated for 20 min on ice, and centrifuged at 155,000 × *g* for 35 min at 4 °C. Supernatants (SP) were lyophilized, and pellets (P) were solubilized in Laemmli buffer for 5 min at 95 °C. Samples were analyzed by immunoblotting.

### Co-localization studies

HCT116 or HeLa cells were seeded in cover glasses and transfected with BiFC constructs of BCL2 and/ or BAX TMDs or with the GFP BCL2 FL proteins, as previously indicated. 16 h post-transfection cells were stained with MitoTracker™ Deep Red FM (Invitrogen #M22426) for mitochondria or ER-ID red assay kit (ENZO technologies ENZ-5106-K500) for ER staining and visualized in a SP8 Leica confocal microscopy. Pearson coefficients were obtained from at least ten different cells and calculated using the Image J 1.54 software with the JACoP (Just Another Co-localization Plugin) plugging.

### Caspase 3/7 activity

HCT116 cells were plated in six-well plates at 250,000 cells/well for 24 h. Cells were then transfected with 0.5 µg of VN BCL2 and VC BAX TMD plasmids or BCL2 FL and BAX FL plasmids using TurboFect (Thermo Scientific™), according to the manufacturer’s instructions. After 16 h of protein expression, cells were harvested, and S100 cytosolic extracts were obtained. Total protein concentration was quantified using the bicinchoninic acid method. For caspase 3/7 kinetics, 60 μg protein was mixed with caspase assay buffer (PBS, 10% glycerol, and 2 mM dithiothreitol) containing 20 μM Ac-DEVD-AFC substrate. Caspase activity was monitored using a Wallac 1420 Workstation (*λ*_exc_ 400 and *λ*_em_ 508 nm).

### Crystal violet staining and viability measurement

HCT116 cells were seeded in 24-well plates at a concentration of 50,000 cells per well and transfected with BCL2 plasmids. After 48 h, the cells were fixed with 4% PFA and stained with a 0.05% crystal violet solution. After capturing images, crystal violet was resuspended in 20% acetic acid, and absorbance at *λ* 580 nm was measured using a Wallac 1420 Workstation.

### Confocal live cell imaging

HeLa or HCT116 cells were transfected in a microscope cover glass (24 mm diameter) stained with Hoechst for nuclei detection, MitoTracker™ Deep Red FM for mitochondria staining, or ER-ID red assay kit for ER staining. Image acquisition used a Leica SP8 confocal microscope.

### Immunofluorescence

HCT116 WT cells and HCT116 BAX KO were seeded at a concentration of 50,000 cells and transfected with BAX TMD or BAX FL plasmids. After 24 h, cells were fixed using PFA 4%, block with 5% BSA in PBS and incubated with primary mouse BAX 6A7 antibody (Invitrogen #MA5-14003) at 1:200 overnight. After several washes, cells were incubated with the secondary antibody goat anti-mouse IgG conjugated Alexa Fluor 488 (#A32723 Thermo Fisher Scientific). Images were obtained using a Leica SP8 confocal microscope.

### Statistical analysis

Graph Pad Prism 10 software was used for statistical analysis. All values represent the mean ± SEM of at least three independent experiments. Statistical significance was determined by one-way ANOVA applying Dunnett’s test and Sidak’s test.

## Results

### Somatic mutations in the BCL2 TMD in cancer patients

Our search of the COSMIC database (https://cancer.sanger.ac.uk/cosmic) identified eight distinct somatic mutations in cancer patients that affect the BCL2 TMD, which impacted various tumor types (including high-incidence tumors such as colon, ovary, or lung cancer) (Fig. 1, Supplementary Table S1). The COSMIC database classifies mutants as pathogenic/non-pathogenic based on a probabilistic score calculated using the FATHMM-MKL algorithm [20], which predicts the functional, molecular, and phenotypic consequences of protein variants using hidden Markov models. This model classified functional scores for mutations with p values above 0.7 as ‘pathogenic’ (http://fathmm.biocompute.org.uk/). While the different BCL2 TMD mutations possessed varying p scores, all lay within the pathogenic range (Fig. 1A). To identify the putative BCL2 TMD interaction surfaces affected by these mutations, we evaluated the spatial distribution of mutant residues on a BCL2 TMD helical wheel projection (https://pss.sjtu.edu.cn/cgi-bin/wheel.cgi?sequence; Fig. 1B). These studies indicated that mutated residues located to distinct spatial positions and could affect different interaction surfaces with other family members, supporting putative differences in the final protein functionality.

### Mutations impacting the BCL2 TMD alter the BCL2 mitochondrial membrane interaction network

The BCL2 TMD interaction network comprises complex homo- and hetero-interaction equilibria between anti- and pro-apoptotic BCL2 family members; therefore, any alterations to these equilibria may impact cell fate. We recently described the ability of BCL2 TMDs to form homo-dimers [17] therefore, we first investigated to what extent mutations affecting the BCL2 TMD affected homo-dimerization.

**Fig. 2:**
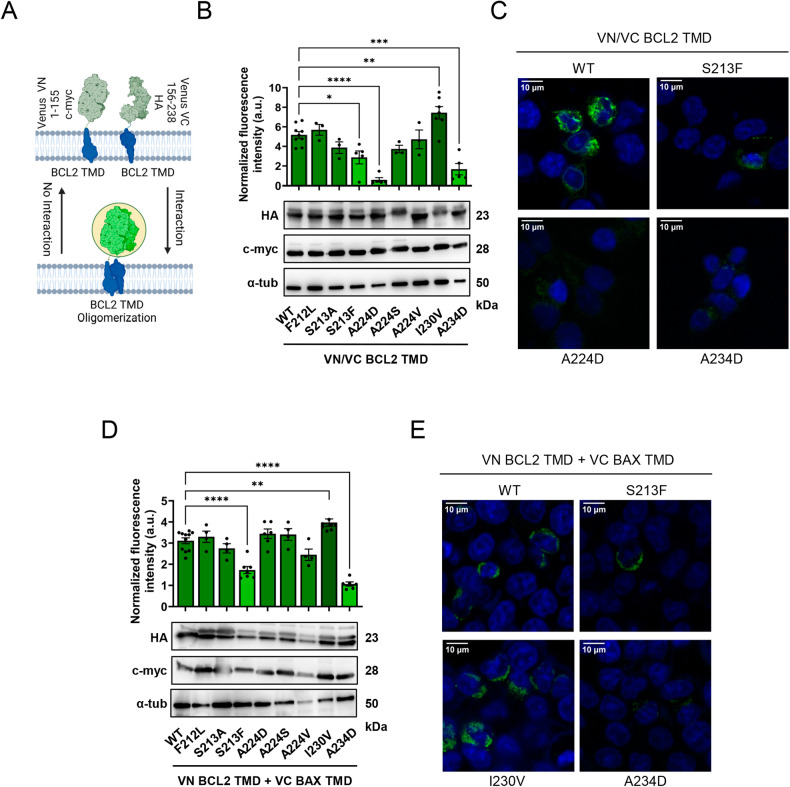
Mutations affecting the BCL2 TMD interfere with oligomerization. **A** Scheme of the engineered BiFC assay analyzing TMD oligomerization through the reconstitution of Venus fluorescence from amino-terminal (VN) or the carboxy-terminal (VC) fragments fused to TMD segments. Constructs include an epitope to analyze levels of expression of the chimeric proteins: c-myc for VN and HA for VC plasmids. **B** Single amino acid mutations impacting the BCL2 TMD disrupt homo-oligomerization. BiFC signal measured in HCT116 cells expressing wild-type (WT) or mutant BCL2 TMDs. Fluorescence intensity levels were normalized by the average of HA and c-myc levels related to tubulin according to quantified immunoblotting. Data represented as mean ± SEM of at least *n* = 3. Significant differences compared to WT BCL2 TMD analyzed using Dunnett’s multiple comparison test (*p < 0.05, ****p < 0.0001). Chimeric protein expression of VN (c-myc) and VC (HA) constructs compared in the lower panel; α-tubulin (α-tub) was used as a loading control. **C** Representative confocal microscopy images of fluorescent signals from the previous graph. Blue signal = Hoechst staining and green signal = Venus reconstitution. Scale bar = 10 µm. **D** Single amino acid mutations disrupt BCL2/BAX TMD oligomerization. Interactions between BCL2 TMD mutants and BAX TMD were analyzed using the BiFC system in HCT116 cells. Data represented as mean ± SEM of at least *n* = 4. Significant differences compared to the WT BCL2 TMD analyzed using Dunnett’s multiple comparison test (**p < 0.01, ***p < 0.001). Chimeric protein expression of VN (c-myc) and VC (HA) constructs were compared in the lower panel; α-tubulin was used as a loading control. **E** Representative confocal microscopy images of fluorescent signals from the previous graph. Blue signal = Hoechst staining and green signal = Venus reconstitution. Scale bar = 10 µm.

To study interactions among BCL2 TMDs, we introduced transmembrane regions into a bimolecular fluorescence complementation (BiFC) system (Fig. 2A) in which the two TMDs under study are fused to either the amino-terminal (VN) or the carboxy-terminal (VC) region of the Venus fluorescent protein (VFP). By employing this BiFC system, we have previously demonstrated that the BCL2 TMD forms homo-oligomers, as compared to the transmembrane domains of control mitochondrial proteins such as TOM20, which are unable to oligomerize [17]. We included c-myc and HA epitopes in the amino-terminal region of the proteins to control the expression levels of the chimera proteins. We co-transfected both BCL2 TMD constructs into HCT116 human colorectal carcinoma cells and followed positive TMD interactions through the reconstitution of VFP and the associated increase in fluorescence intensity. The BiFC BCL2 TMD homo-interaction assay confirmed that three out of the eight mutants analyzed induced a significant reduction in homo-oligomerization (S213F, A224D, and A234D) compared to the wild-type (WT) BLC2 TMD (Fig. 2B). Interestingly, these mutants have protein expression levels comparable to the wild-type protein (Fig. 2B, lower panel), indicating that the decrease in fluorescence observed in the BiFC assays (Fig. 2C) results from altered TMD interactions.

The BCL2 TMD also hetero-oligomerizes with the TMD of the pro-apoptotic BAX protein, a mechanism that controls cell death by preventing the formation of pores in the outer mitochondrial membrane [17]. For this reason, we explored the impact of BCL2 TMD mutants on this hetero-interaction using the BiFC assay. Our results revealed that the S213F and A234D mutations induced a significant decrease in the BCL2/BAX TMD hetero-interaction compared to the WT BLC2 TMD (Fig. 2D, E); however, the I230V mutation induced a significant increase in the BCL2/BAX TMD hetero-interaction compared to the WT BLC2 TMD, probably due to a tighter packing interface (Fig. 2D, E). Again, we found that protein expression levels did not significantly vary among samples (Fig. 2D, lower panel**)**. To address potential interferences from the HCT116 cellular background affecting the behavior of the BCL2 TMD mutants, we investigated the homo- and hetero-oligomerization of BCL2 TMD with BAX TMD in the HeLa cervix adenocarcinoma cell line. This analysis confirmed that the behavior of the mutants was consistent across both cell lines (Supplementary Fig. S1).

Interestingly, the differences observed in the impacts of these mutants in homo- and hetero-oligomerization assays indicate that the interaction surface of the BCL2 TMD homo-dimer partially overlaps but does not precisely match the BCL2/BAX TMD hetero-dimerization interface. Mutants altering the BCL2 TMD hetero-interactions with pro-apoptotic BAX remain particularly interesting due to the possible impacts on cancer cell survival and therapeutic resistance.

### BCL2 TMD mutants preserve their membrane insertion capacity

**Fig. 3:**
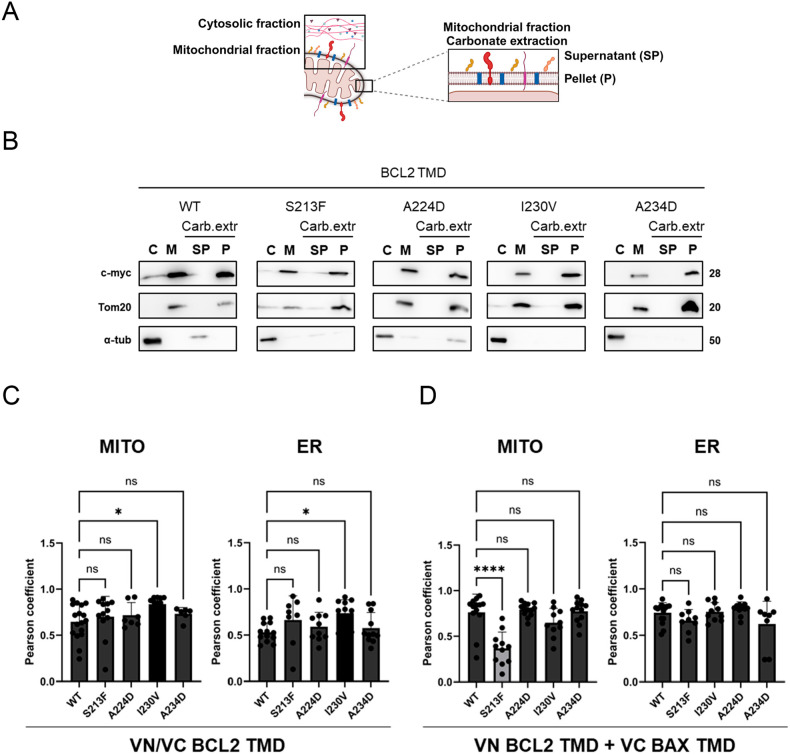
BCL2 TMD mutants insert into the mitochondrial membrane. **A** Schematic representation of the subcellular fractionation procedure to confirm protein membrane insertion. **B** Subcellular fractionation and carbonate extraction (Carb. extr) of mitochondria from HCT116 cells expressing BCL2 TMD mutants. Locations are controlled using Tom20 (mitochondrial fraction, M) and α-tubulin (cytosol, C). The mitochondrial fraction was treated with a sodium carbonate solution to remove loosely attached membrane proteins (soluble fraction, SP; membrane proteins, P). **C** Subcellular distribution of homo-oligomers of BCL2 TMD mutants in the mitochondria and ER. Representation of Pearson correlation coefficients between the BCL2 TMD constructs and Mitotracker or ER tracker. **D** Subcellular distribution of hetero-oligomers of BCL2 TMD mutants with BAX TMD in the mitochondria and ER. Representation of Pearson correlation coefficients between the BCL2 TMD constructs and Mitotracker or ER tracker. Data represented as mean ± SEM of at least *n* = 3. Significant differences compared to WT BCL2 TMD analyzed using Dunnett’s multiple comparison test (*p < 0.05, ****p < 0.0001).

Before drawing firm conclusions regarding the consequences of these mutations on TMD oligomerization, we studied if variations in the BCL2 TMD surface introduced by mutations could interfere with protein insertion within the membrane. For subsequent studies we focused only on those mutants where oligomerization is affected. We performed subcellular fractionation assays with HCT116 cells to discount alterations to TMD insertion defects as the reason for decreased oligomerization observed in BiFC assays (Fig. 3A). Subcellular fractionation of the S213F, A224D, I230V, and A234D mutants confirmed that these BCL2 proteins become appropriately located in the mitochondria-enriched heavy membrane fraction (Fig. 3B; compare membrane fraction M with cytosolic fraction C). We used the characterization of Tom20 and α-tubulin protein levels (mitochondrial membrane and cytosolic markers, respectively) to confirm proper fractionation.

We next employed carbonate extraction to eliminate non-integral membrane-associated proteins and corroborate the proper transmembrane insertion of mutant BCL2 proteins (Fig. 3A). The results of this assay (Fig. 3B; compare supernatant fraction SP with integral membrane fraction P) confirmed that all BCL2 TMD mutants became inserted as for the wild-type protein, supporting the fact that the differences in oligomerization derived from interference within the packing interfaces.

Nevertheless, as BCL2 is located both in the ER and the mitochondria [21], we wondered if the endomembrane distribution could change as a consequence of the transmembrane mutations. Co-localization studies of the BCL2 TMD homo- and BCL2/BAX TMDs hetero-dimers (Fig. 3C, D, Supplementary Fig. S4) did not show significant differences in organelle distribution among the mutants, with only a slight increase in endomembrane co-localization in the case of the BCL2 I230V TMD mutant (ER and mitochondria), and a mitochondrial co-localization decrease in BCL2 S213F TMD/BAX TMD hetero-oligomers.

Overall, we expect that the interaction balance between homo- and hetero-dimerization within the membrane and the changes in endomembrane distribution would determine whether mutations affecting the BCL2 TMD positively or negatively affect the ability of BCL2 to protect against cell death.

### BCL2 TMD mutants may display defective responses to pro-apoptotic stimuli

We next studied mutants in a cell death context to fully understand the correlation between BCL2 TMD mutations, oligomerization ability, and functional consequences. We first studied the effect of mutations on isolated TMDs, which allows us to distinguish effects that affect TMD-cell death modulation ability from those that affect global protein behavior. Previous studies from our group demonstrated that BAX TMD expression induced apoptosis in HCTT116 cells [17]. The induction of cell death observed in this study is independent of endogenous BAX expression, as it does not activate endogenous BAX and also occurs in HCT116 DKO cells (Supplementary Fig. S2). Interestingly, this cell death is counteracted by the BCL2 TMD, suggesting that it is not a non-specific event caused by transmembrane overpopulation, but most probably results from the activation of downstream events in the apoptosis pathway. In this sense, we have previously demonstrated that BAX TMD peptides induce the release of cytochrome c in isolated mitochondria [22].

We reasoned that the mutants affecting BCL2/BAX hetero-interaction could interfere with the ability to modulate cell death; therefore, we explored the potential for BCL2 TMD mutants to preserve their ability to prevent BAX TMD-induced apoptosis.

**Fig. 4:**
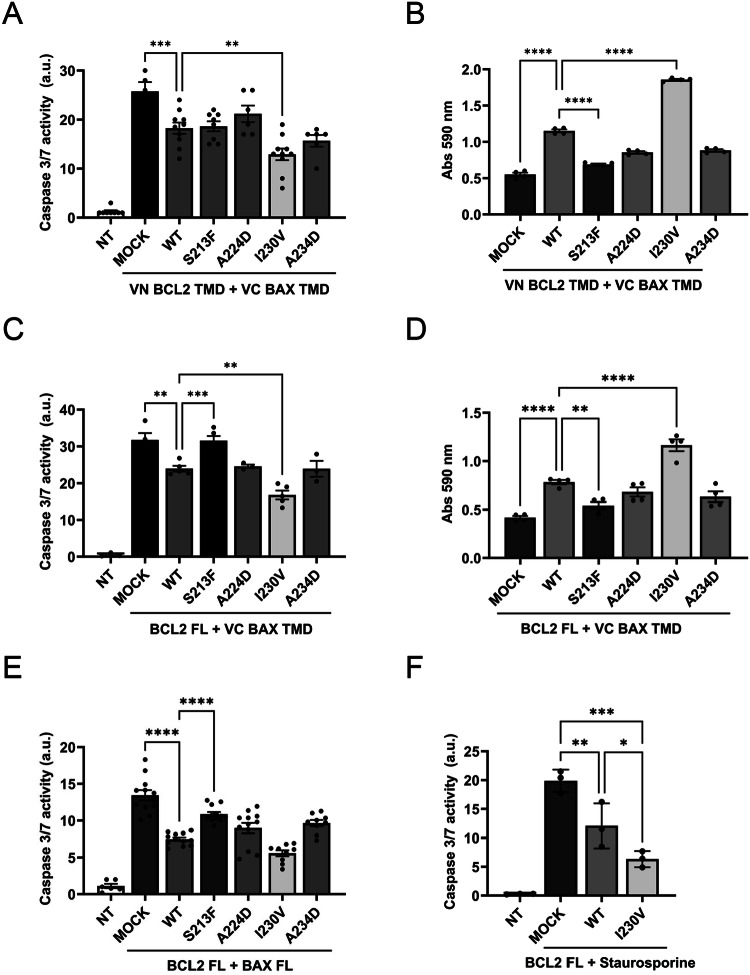
Single amino acid mutations in the BCL2 TMD impair BCL2 apoptotic function. **A** The anti-apoptotic activity of BCL2 TMD mutants in response to BAX TMD-induced cell death measured by caspase 3/7 activity in HCT116 cells co-transfected with VN BCL TMD constructs. Data represented as mean ± SEM of at least *n* = 6. Significant differences compared to BCL2 TMD WT analyzed using Šídák’s multiple comparison tests (**p < 0.01, ***p < 0.001). NT = non-transfected; MOCK = cells transfected with empty VN plasmid. **B** Quantification of HCT116 cell viability measured by crystal violet staining. Data represented as mean ± SEM, *n* = 4. Significant differences compared to the BCL2 TMD WT analyzed using Šídák’s multiple comparison tests (****p < 0.0001). **C** The anti-apoptotic activity of BCL2 FL mutants in response to BAX TMD-induced cell death measured by caspase 3/7 activity in HCT116 cells co-transfected with BCL2 FL mutants. Data represented as mean ± SEM of at least *n* = 3. Significant differences compared to the BCL2 FL WT analyzed using Šídák’s multiple comparison tests (**p < 0.01, ***p < 0.001). NT = non-transfected; MOCK = cells transfected with pCDNA3.1 empty plasmid. **D** Quantification of HCT116 cell viability measured by crystal violet staining. Data represented as mean ± SEM, n = 4. Significant differences compared to the BCL2 TMD WT analyzed using Šídák’s multiple comparison tests (**p < 0.01, ****p < 0.0001). **E** The anti-apoptotic activity of BCL2 FL mutants in res*p*onse to BAX FL-induced cell death measured by caspase 3/7 activity in HCT116 cells co-transfected with BCL2 FL mutants. Data represented as mean ± SEM of at least n = 3. Significant differences compared to the BCL2 FL WT analyzed using Šídák’s multiple comparison tests (**p < 0.01, ***p < 0.001). NT = non-transfected; MOCK = cells transfected with pCDNA3.1 empty plasmid. **F** Caspase 3/7 activity was measured in HCT116 cells transfected with BCL2 FL WT or I230V mutant and treated with 1 µM staurosporine for 24 h. Data represented as mean ± SEM (*n* = 3; *p < 0.05, **p < 0.01, ***p < 0.001). NT = non-transfected/non-treated, MOCK = cells transfected with pCDNA3.1 empty plasmid.

Our results demonstrated that the expression of the BCL2 TMD I230V mutant, which inserts appropriately in the membrane and enhances the interaction with BAX TMD, significantly enhanced the inhibition of apoptosis compared to WT BCL2 TMD, as measured by a decrease in caspase 3/7 activity (Fig. 4A). As expected, this effect correlated with a significant increase in cell survival, as evaluated by positive crystal violet staining (Fig. 4B). The decreased hetero-interactions observed for the BCL2 S213F and A234D mutants (Fig. 2D) did not directly translate into a significant loss of apoptosis inhibition capability in the context of isolated TMD interactions; however, we observed a tendency to decreased cell survival in the case of the A234D mutant and a significant decrease in the case of the S213F mutant (Fig. 4B, Supplementary Fig. S3A), indicating the loss of BAX TMD-induced cell death counteracting ability.

We also observed comparable results when counteracting BAX TMD-induced apoptosis by mutations introduced into the full-length BCL2 (BCL2 FL) protein (Fig. 4C). BCL2 FL I230V counteracted caspase-3 activation significantly better than the wild-type protein, while the BCL2 FL S213F mutant lost the ability to inhibit apoptosis observed with BCL2 FL WT expression (Fig. 4C). As expected, these alterations to apoptosis correlated with a significant increase in cell viability in cells transfected with BCL2 FL I230V compared to wild-type BCL2 FL, while a decrease in viability occurred in cells transfected with BCL2 FL S213F (Fig. 4D, Supplementary Fig. S3B). The same tendency was observed when BAX FL-induced cell death was counteracted by BCL2 FL mutants (Fig. 4E).

**Fig. 5:**
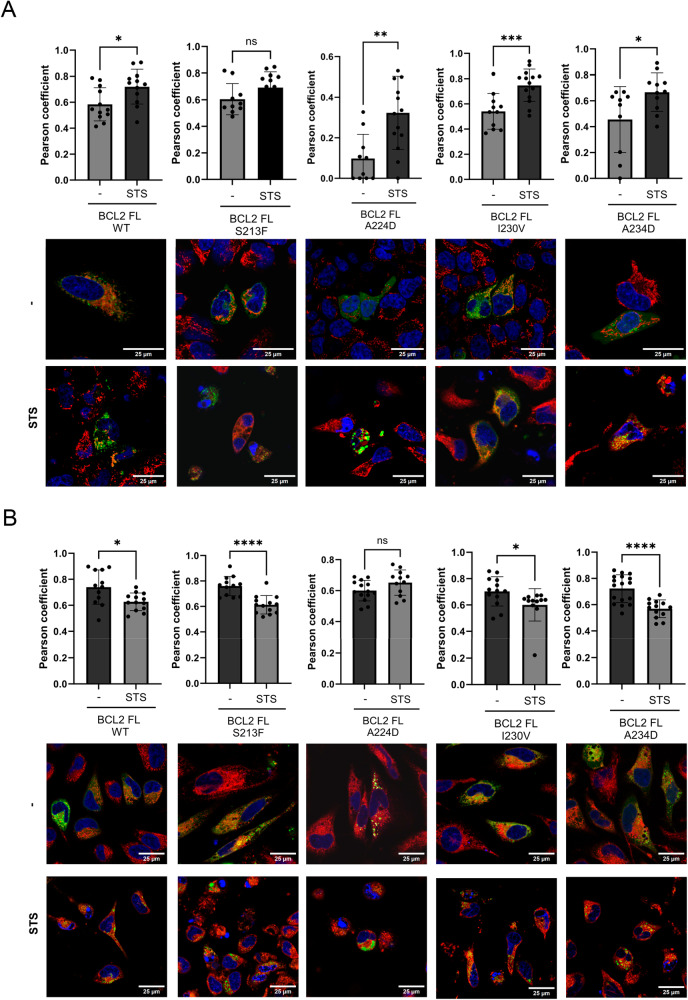
Characterization of subcellular distribution of BCL2 FL mutants in healthy and apoptotic cells. Representation of Pearson correlation coefficients between the GFP BCL2 FL constructs (green) and Mitotracker (red) (**A**) or ER-tracker (red) (**B**) in healthy (−) and staurosporine (STS; 1 μM) HeLa treated cells. Representative confocal images are depicted below the graphs. Cell nuclei are stained with Hoescht (blue). Data represented as mean ± SEM of at least *n* = 3. Significant differences compared to WT BCL2 FL analyzed using Dunnett’s multiple comparison test (*p < 0.05, **p < 0.01, ***p < 0.001, ****p < 0.0001). Scale bar = 25 µm.

To study if the subcellular localization of the BCL2 FL protein became affected by TMD mutations, we introduced them into a GFP-BCL2 FL fusion expression construct and transfected them into HeLa cancer cells. HeLa cells were selected to facilitate mitochondria visualization and corroborate mutant behavior in a different cancer cellular model. Our results revealed a distribution compatible with a mitochondrial and ER location (Fig. 5). Cells with higher expression of GFP-BCL2 A224D displayed clustering behavior, which probably induces BAX sequestration given the maintenance of their cell recovery ability. Interestingly, the induction of cell death by staurosporine led to an increase in the co-localization of BCL2 FL protein with mitochondria and a decrease in the ER (Fig. 5A, B), while the behavior is more erratic in isolated TMDs (Supplementary Fig. S4). This phenomenon was observable in all cases except for the BCL2 FL S213F mutant, where the increase in mitochondrial localization upon apoptosis induction is diminished. This result aligns with the observed reduction in TMD hetero-oligomerization and the lower inhibition capability of apoptosis for this mutant.

With a view of putative cancer patient resistance to therapy, I230V represents the most interesting mutation, given its greater capacity to inhibit cell death; therefore, we evaluated the effect of I230V on chemotherapy-induced cell death. Staurosporine, a broad-spectrum inhibitor of protein kinases that functions in an ATP-competitive manner, causes cytotoxicity in several human cancer cells, including colon cancer cells [23–25]. As expected, BCL2 FL WT expression protected from staurosporine-induced cell death in HCT116 cells; however, I230V expression significantly reduced staurosporine-induced apoptosis compared to WT BCL2 TMD (Fig. 4F). The same tendency is observed in cell death induced by the specific BCL2 inhibitor, Venetoclax, where cells overexpressing BCL2 FL I230V exhibit lower caspase-3 activity compared to those overexpressing BCL2 FL WT (Supp Fig. S5). This data indicates that the BCL2 FL I230V somatic mutant behaves in an “over-protective” manner, reducing the effect of cell death stimuli to interfere with pro-apoptotic therapies.

In conclusion, these results demonstrate that cancer-associated mutations in the BCL2 TMD may affect BCL2 functionality and contribute to the regulation of cell death.

## Discussion

The BCL2 family of proteins plays essential roles in controlling cell survival [26]; therefore, their altered expression and the appearance of BCL2 somatic mutations are associated with tumor progression, drug resistance, and disease recurrence [27–30]. Understanding the molecular changes that mutations affecting these proteins trigger remains essential to provide cancer patients with new treatment strategies. Previous studies have demonstrated that specific mutations, mainly those affecting cytosolic domains, correlate with a marked increase in cellular transformation and accelerated cancer patient death [12].

This study focused on somatic mutations affecting the anti-apoptotic BCL2 protein, particularly relatively unexplored low-frequency mutations affecting the TMD. Knowledge regarding the BCL2 TMD interactions remains limited due to the experimental difficulty in working with membrane proteins; however, recent technological advancements have led to growth in the field [31–34]. TMDs exert relevant functions in the modulation of apoptosis, such as the retrotranslocation of the pro-apoptotic protein BAX from the mitochondria to the cytosol to avoid cell death [17, 35]. Our group recently demonstrated that the interaction of BCL2 and BAX through their TMDs contributes to the modulation of cell death [17]; therefore, phenotyping mutations affecting these TMDs could have relevance for cancer cell survival.

In this study, we investigated how mutations impacting the TMDs of BCL2 affect homo- and hetero-dimerization with pro-apoptotic family members and how they alter anti-apoptotic protein functionality. Our results demonstrated that while some mutations have no evident consequences on protein oligomerization, mutations such as S213F or I230V interfere with TMD oligomerization, which generates proteins with differing cell viability preservation abilities. In the case of the BCL2 TMD I230V mutant (found in a metastatic lung adenocarcinoma patient), we observed increased hetero-oligomerization with the BAX TMD, rendering a protein that confers increased resistance to pro-apoptotic stimuli (Fig. 4F). Concerning patient treatment selection, identifying the I230V mutation could justify higher drug dosages or a BAX-independent cell death-inducing treatment to provoke an adequate therapeutic response. Conversely, S213F (found in a metastatic melanoma patient) displays a lower BAX TMD binding ability and a decrease in BCL2/ BAX mitochondrial localization (Fig. 3D), thereby providing a lower level of protection from cell death (Fig. 4C, D). In this case, the patient’s response to drugs inducing BAX-dependent apoptosis should be more significant.

Notably, the presence of the A234D mutation induced a slight decrease in hetero-oligomerization with the BAX TMD, which did not translate into changes in apoptosis. Insertion of polar ionizable residues within the membrane is an uncommon event; however, when this occurs, the penalty of exposing the polar sidechains to the environment increases oligomer stability [36]. This energetic penalty could provoke changes in the reversibility of the oligomer equilibrium, finally rendering a protein that behaves similarly to the wild type in terms of apoptotic modulation.

Overall, our results contribute to BCL2 mutational studies with additional experimental data about protein behavior with regard to cancer cell survival and provide new insight to guide decisions for optimal patient treatment, distinguishing driver mutations from passenger mutations. Algorithm-based predictions possess immense potential in terms of global understanding of tumor behavior and have recently led to tremendous advances in the biomedical field [15, 37]. As demonstrated in this study, combining predictive models with a detailed experimental understanding of cancer somatic mutants may provide relevant data for developing personalized treatments that would feed future algorithms to obtain more accurate predictions.

### Supplementary information

Supplementary Information

## Data Availability

Data are available via application to the corresponding author.
