# Supplementary material for: Phenotyping of cancer-associated somatic mutations in the BCL2 transmembrane domain
Source: Oncogenesis. 2024 Apr 26;13(1):14. doi: 10.1038/s41389-024-00516-3 (PMC11052995; doi:10.1038/s41389-024-00516-3)
Supplement: Supplementary file 1 — Supplementary Information [file 41389_2024_516_MOESM1_ESM.pdf]

# Supplementary Information

**Phenotyping of cancer-associated somatic mutations in the BCL2 transmembrane domain**

Diego Leiva[1], Estefanía Lucendo[1], Alicia Belén García-Jareño[1], Mónica Sancho[1,#], and Mar Orzáez[1,#]

[1]Targeted Therapies on Cancer and Inflammation Laboratory, Centro de Investigación Príncipe Felipe, Valencia, Spain

[#]Corresponding authors: msancho@cipf.es ; morzaez@cipf.es

**Supplementary Table I**. Data of the patient samples where BCL-2 TMD mutations were found.

| **MUTANT** | **F212L** | **S213A** | **S213F** | **A224D** | **A224S** | **A224V** | **I230V** | **A234D** |
|---|---|---|---|---|---|---|---|---|
| Attribute | Value | Value | Value | | Value | Value | Value | Value |
| Number of Samples Per Patient | 1 | 1 | 1 | | | | 1 | 1 |
| Overall Survival (Months) | 16.1 | 3.1 | 10.1 | | | | 21.7 | |
| Overall Survival Status | 0:LIVING | 1:DECEASED | 0:LIVING | | | 0:LIVING | 0:LIVING | 1:DECEASED |
| Patient's Vital Status | ALIVE | DECEASED | ALIVE | | 10 years free survival | | ALIVE | DECEASED |
| Sex | Male | Male | Male | Male | Male | Female | Female | Female |
| Smoking History | Prev/Curr Smoker | Prev/Curr Smoker | Prev/Curr Smoker | | Heavy Smoker | | Prev/Curr Smoker | Never |
| Attribute | P-0004865-T01-IM5 | P-0006499-T01-IM5 | P-0009189-T01-IM5 | | LUAD-S01405 | SP82900 | P-0002957-T01-IM3 | P-0002136-T01-IM3 |
| Mutation Count | 146 | 12 | 67 | | 301 | 1246 | 20 | 7 |
| Fraction Genome Altered | 0.0000 | 0.1660 | 0.0215 | | | | 0.6923 | 0.5933 |
| Cancer Type | Colorectal Cancer | Mature B-Cell Neoplasms | Melanoma | Liver neoplasm | Non-Small Cell Lung Cancer stage IIA | Melanoma | Non-Small Cell Lung Cancer | Ovarian Cancer |
| Cancer Type Detailed | Colon Adenocarcinoma | Diffuse Large B-Cell Lymphoma, NOS | Cutaneous Melanoma | | Lung Adenocarcinoma | Melanoma malignant | Lung Adenocarcinoma | Mixed Ovarian Carcinoma |
| DNA Input | 250 | 250 | 160.9 | | | | 250 | 250 |
| Matched Status | Matched | Unmatched | Matched | | Matched | | Matched | Matched |
| Oncotree Code | COAD | DLBCLNOS | SKCM | | LUAD | MEL | LUAD | MXOV |
| Primary Tumor Site | Ascending Colon | Lymph node | Skin | | | SKIN | Lung | Ovary |
| Sample Class | Tumor | Tumor | Tumor | | | Tumor/Metastasis | Tumor | Tumor |
| Sample Collection Source | In-House | In-House | In-House | COSS2634306 | CLCGP / MPI | SA432539 | Outside (metastasis site lymph node) | Outside |
| Sample coverage | 838 | 1037 | 254 | | | | 349 | 402 |
| Sample Type | Primary | Primary | Primary | tumor | | | Metastasis | Primary |
| Somatic Status | Matched | Unmatched | Matched | | Matched | | Matched | Matched |
| Specimen Preservation Type | DNA | FFPE | FFPE | | | | FFPE | FFPE |
| Specimen Type | Resection | Biopsy | Biopsy | | | | Biopsy | Resection |
| Tumor Purity | 50 | 60 | 80 | | | | 30 | 60 |

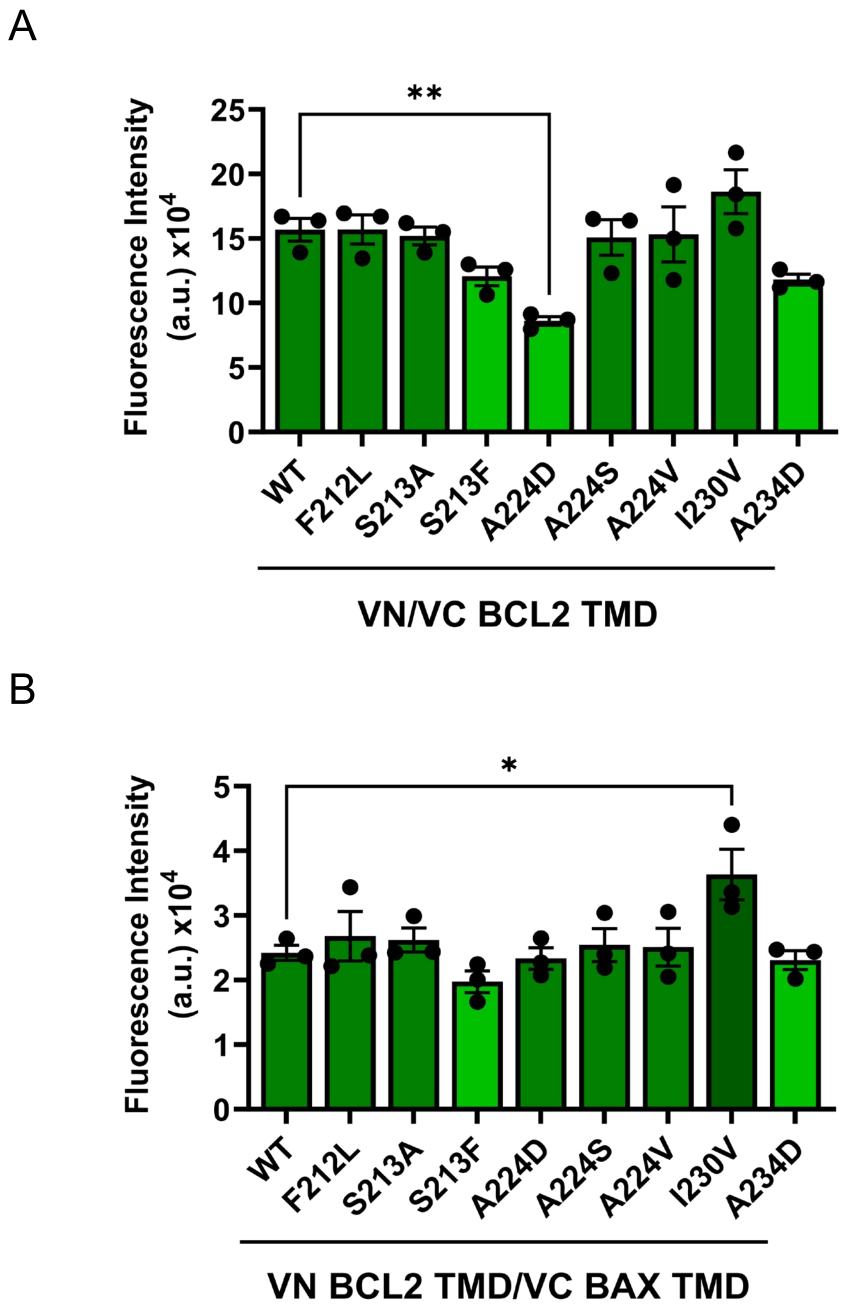


**Supp Figure 1. Mutations affecting the BCL2 TMD interfere with oligomerization in HeLa cells.** **A** Single amino acid mutations impacting the BCL2 TMD disrupt homo-oligomerization. BiFC signal measured in HeLa cells expressing wild-type (WT) or mutant BCL2 TMDs. **B** Single amino acid mutations disrupt BCL2/BAX TMD oligomerization. Interactions between BCL2 TMD mutants and BAX TMD were analyzed using the BiFC system. Data represented as mean ± SEM of at least n = 3. Significant differences compared to the WT BCL2 TMD analyzed using Dunnett's multiple comparison test (*p<0.05, **p<0.01).

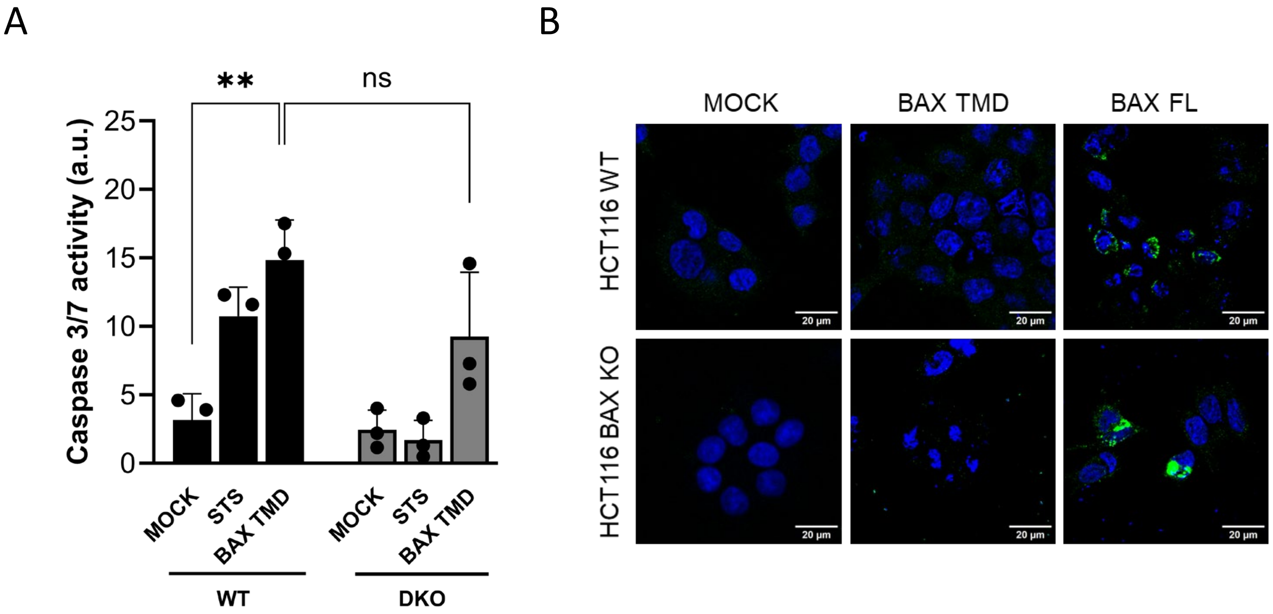


**Supp Figure 2. BAX TMD induces apoptosis in WT and BAX/BAK DKO HCT116 cells. A** Cell death measured by caspase 3/7 activity in HCT116 cells transfected with BAX TMD constructs in WT and BAX-/-BAK-/- cells (DKO). Cells treated with staurosporine (STS) 1 µM were included as positive control of apoptosis induction. MOCK refers to empty vector. Data represented as mean ± SEM of n = 3. Significant differences compared to the MOCK or BAX TMD analyzed using Šídák's multiple comparison tests (**p<0.01). **B** The transgenic BAX TMD do not displace the endogenous BAX protein to induce activation. Immunofluorescence of active BAX (clone 6A7; green signal) in HCT116 WT and BAX KO, overexpressing either BAX TMD or BAX FL, (MOCK = empty vector). Blue signal corresponds to nuclei, scale bar is 25 µm.

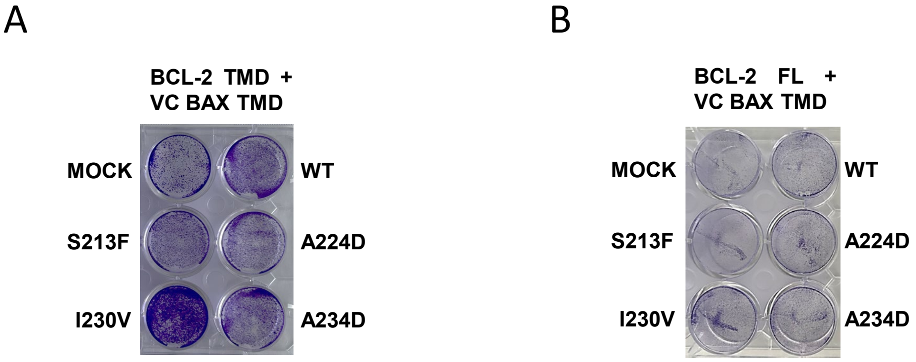


**Supp Figure 3. Representative images of crystal violet staining in cell viability studies showing that some single amino acid mutations in the BCL2 TMD impair BCL2 apoptotic function. A** HCT116 cells were transfected with BCL2 TMD mutants and BAX TMD. After 48 h, cells were fixed and stained with crystal violet. **B** HCT116 cells transfected with BCL2 FL mutants and BAX TMD and stained with crystal violet following the same procedure as A.

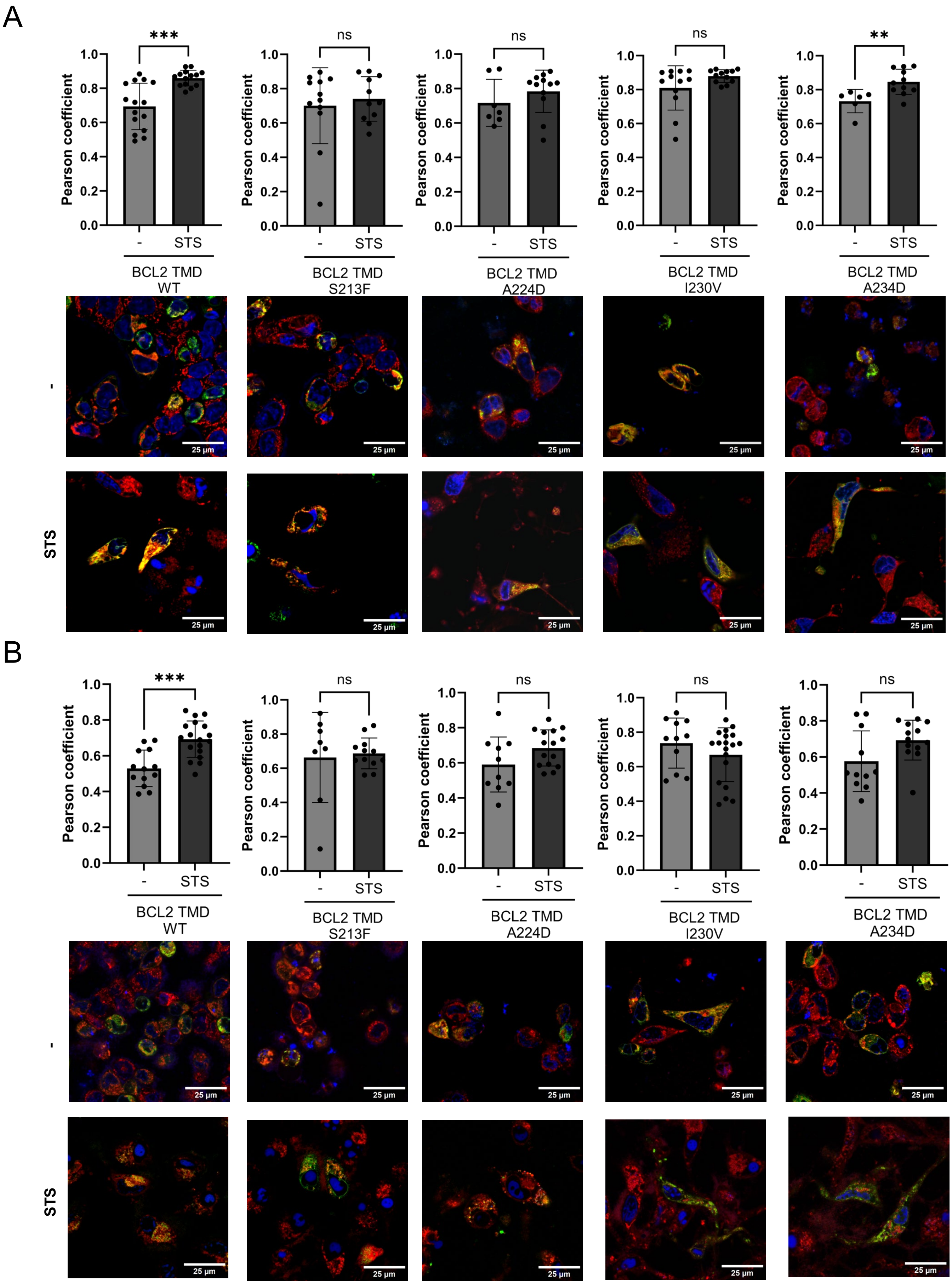


**Supp Figure 4. Characterization of subcellular distribution of BCL2 TMDs in healthy and apoptotic cells.** Representation of Pearson correlation coefficients between the BiFC BCL2 TMD constructs (green) and mitotracker (red) **(A)** or ER-tracker (red) **(B)** in healthy (-) and staurosporine (STS; 1µM) HCT116 treated cells. Representative confocal images are depicted below the graphs. Cell nuclei are stained with Hoescht (blue). Data represented as mean ± SEM of at least n = 3. Significant differences compared to WT BCL2 TMD analyzed using Dunnett's multiple comparison test (**p<0.01, ***p<0.001). Scale bar is 25 µm.

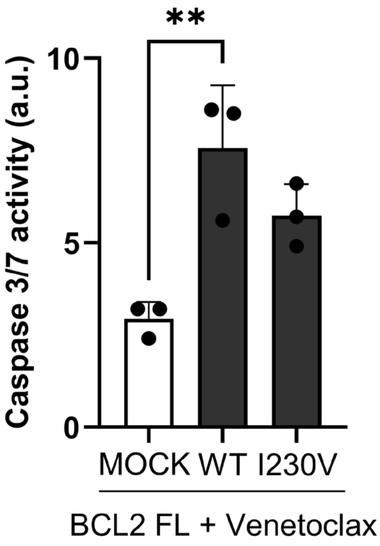


**Supp Figure 5. Cell death induced by Venetoclax in cells overexpressing BCL2 FL I230V is lower than in WT**. Caspase-3/7 activity measured in HCT116 cells transfected with BCL2 FL WT or I230V and treated with 25 µM Venetoclax for 24 h. Data represented as mean ± SEM (n = 3, **p<0.01). MOCK refers to cells transfected with pCDNA3.1 empty plasmid.
